# Supplementary material for: DMRforPairs: identifying Differentially Methylated Regions between unique samples using array based methylation profiles
Source: BMC Bioinformatics. 2014 May 15;15:141. doi: 10.1186/1471-2105-15-141 (PMC4046028; doi:10.1186/1471-2105-15-141)
Supplement: Additional file 2 — DMRforPairs output for the comparison of A431-MCF7 and NA17018-NA17105. Please start from the HTML files in each folder. Available via the BMC Bioinformatics website. [file 1471-2105-15-141-S2.zip › 1394847754114233_MOESM2_ESM/A431_MCF7/figures/23133.pdf]

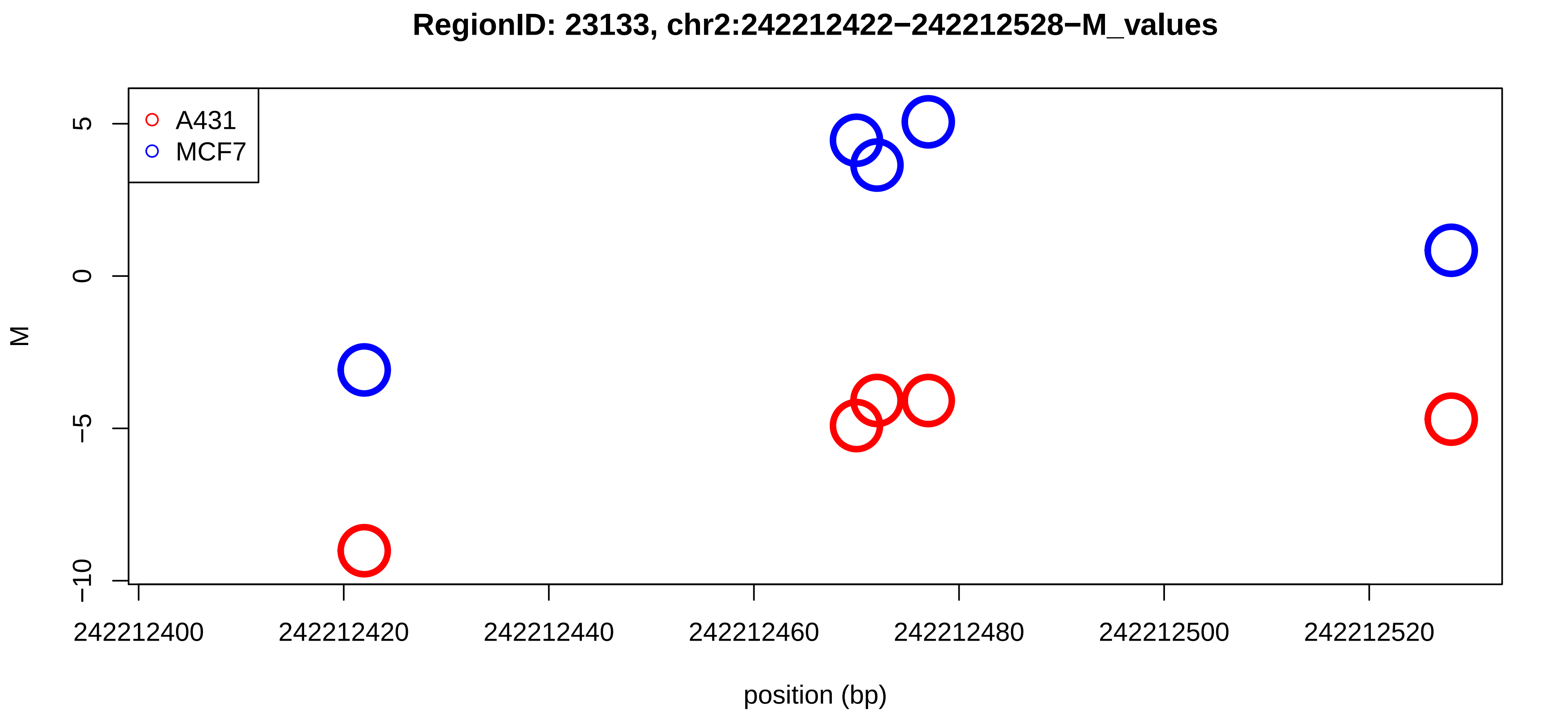
RegionID: 23133, chr2:242212422−242212528−M_values
A431
MCF7
M
5
0
−5
−10
242212400
242212420
242212440
242212460
242212480
242212500
242212520
position (bp)

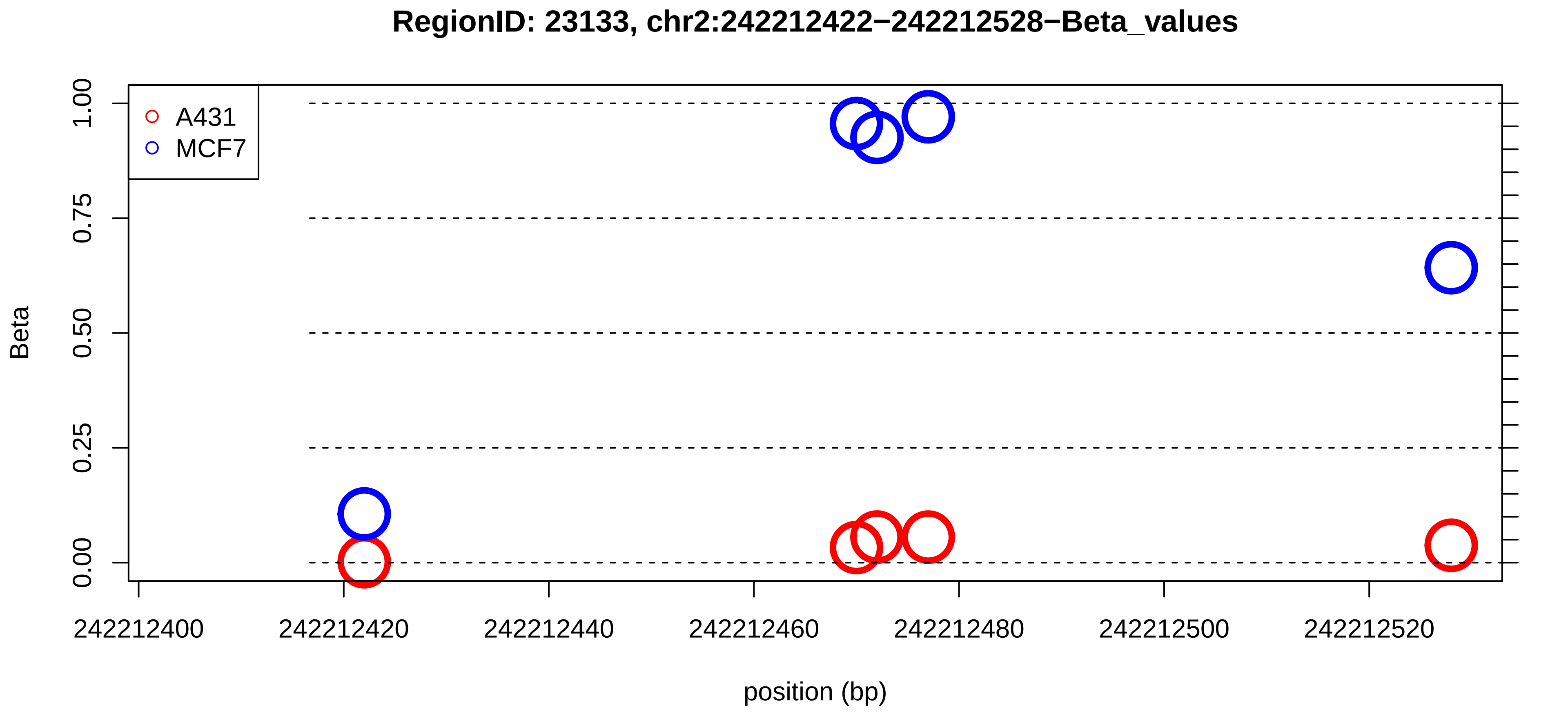
RegionID: 23133, chr2:242212422−242212528−Beta_values
A431
MCF7
Beta
1.00
0.75
0.50
0.25
0.00
242212400
242212420
242212440
242212460
242212480
242212500
242212520
position (bp)

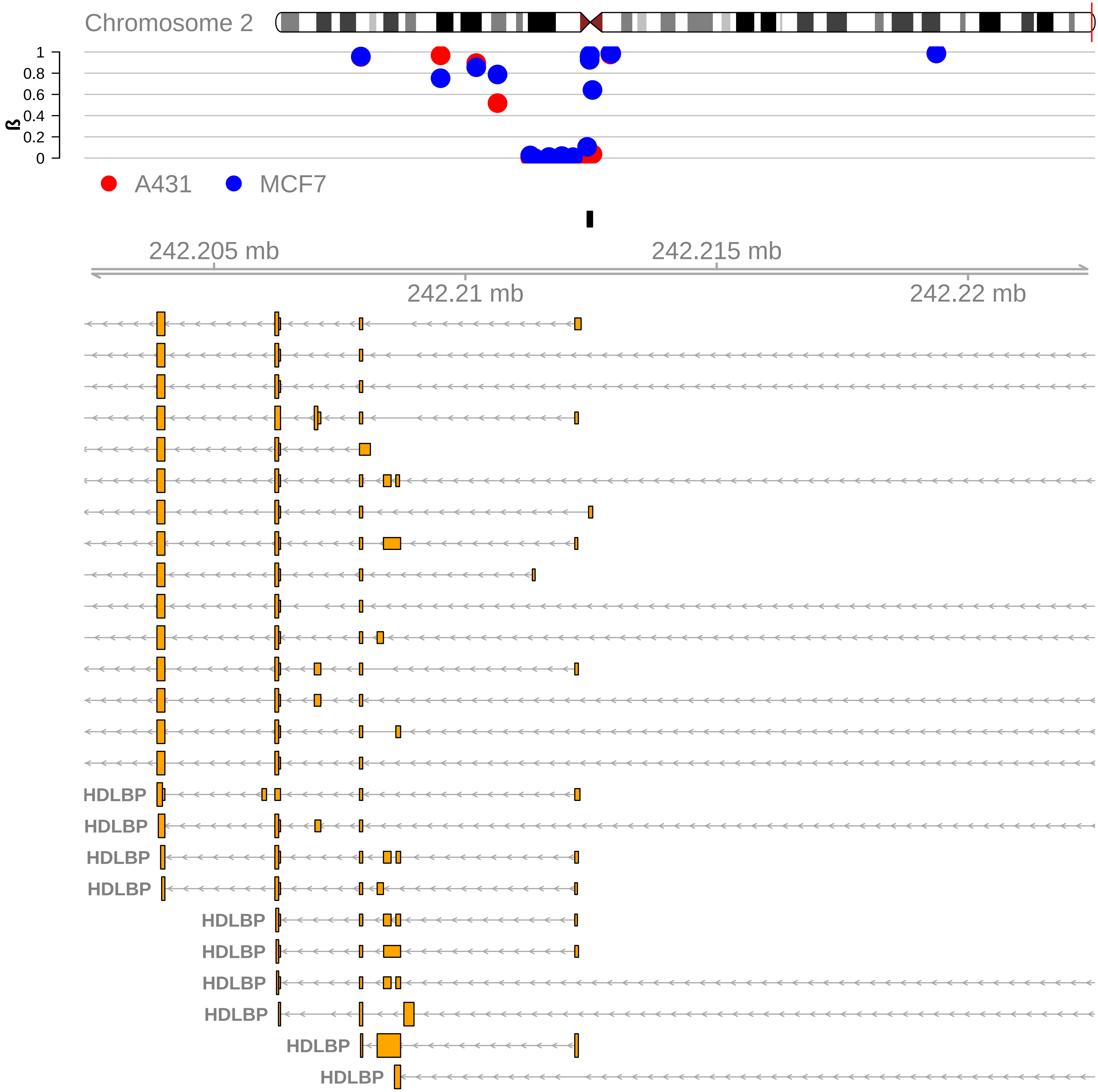

Chromosome 2
ß
1
0.8
0.6
0.4
0.2
0
A431
MCF7
242.205 mb
242.215 mb
242.21 mb
242.22 mb
HDLBP
HDLBP
HDLBP
HDLBP
HDLBP
HDLBP
HDLBP
HDLBP
HDLBP
HDLBP
